# Dispersion and Homogeneity of MgO and Ag Nanoparticles Mixed with Polymethylmethacrylate

**DOI:** 10.3390/polym15061479

**Published:** 2023-03-16

**Authors:** Awder Nuree Arf, Fadil Abdullah Kareem, Sarhang Sarwat Gul

**Affiliations:** 1 Department of Pedodontics, Orthodontics and Preventive Dentistry, College of Dentistry, University of Sulaimani, Sulaymaniyah 46001, Iraq; awder.arf@univsul.edu.iq (A.N.A.); fadil.kareem@univsul.edu.iq (F.A.K.); 2 Department of Periodontics, College of Dentistry, University of Sulaimani, Sulaymaniyah 46001, Iraq; 3 Presidency of Sulaimani Polytechnic University, Sulaymaniyah 46001, Iraq

**Keywords:** MgO, Ag, nanoparticles, PMMA, dispersion, homogeneity

## Abstract

This study aims to examine the impact of the direct and indirect mixing techniques on the dispersion and homogeneity of magnesium oxide (MgO) and silver (Ag) nanoparticles (NPs) mixed with polymethylmethacrylate (PMMA). NPs were mixed with PMMA powder directly (non-ethanol-assisted) and indirectly (ethanol-assisted) with the aid of ethanol as solvent. X-ray diffraction (XRD), energy-dispersive X-ray spectroscopy (EDX), and scanning electron microscope (SEM) were used to evaluate the dispersion and homogeneity of MgO and Ag NPs within the PMMA-NPs nanocomposite matrix. Prepared discs of PMMA-MgO and PMMA-Ag nanocomposite were analyzed for dispersion and agglomeration by Stereo microscope. XRD showed that the average crystallite size of NPs within PMMA-NP nanocomposite powder was smaller in the case of ethanol-assisted mixing compared to non-ethanol-assisted mixing. Furthermore, EDX and SEM revealed good dispersion and homogeneity of both NPs on PMMA particles with ethanol-assisted mixing compared to the non-ethanol-assisted one. Again, the PMMA-MgO and PMMA-Ag nanocomposite discs were found to have better dispersion and no agglomeration with ethanol-assisted mixing when compared to the non-ethanol-assisted mixing technique. Ethanol-assisted mixing of MgO and Ag NPs with PMMA powder obtained better dispersion, better homogeneity, and no agglomeration of NPs within the PMMA-NP matrix.

## 1. Introduction

Polymers composites play an important role in dentistry, with a wide range of clinical implementations in both restorative and prosthodontics dentistry [1]. Polymers have some distinctive features, such as high strength-to-weight ratios, resistance to corrosion, and a lack of conductivity, which are not available in other types of materials [2]. One of the commonly used polymers in dentistry is poly methyl methacrylate (PMMA), which is available in heat polymerized and self-polymerized acrylic resin forms [3]. PMMA has several advantages that make it the polymer most commonly used as denture base material, such as ease of processing, low cost, light weight, stability in the oral cavity, and esthetic properties [4]. Furthermore, PMMA-based bone cement is a biomaterial that has been used over the last 50 years to stabilize hip and knee implants or as a bone filler [5]. However, PMMA has some drawbacks, including poor surface properties, such as poor wear resistance and porosity, which facilitate the initial adhesion of micro-organisms [6] and weak mechanical properties regarding impact and flexural strengths [7]. On the other hand, prosthesis within the oral cavity is associated with a decrease in the cleansing effect of the tongue and salivary flow, as well as the enhanced formation and deposition of biofilms on both prosthetics and adjacent mucosa [8].

Structural modifications to the composition of PMMA by addition of fillers might enhance the resin’s mechanical [9] and antimicrobial properties [10]. Nanoparticles (NPs) are one type of such fillers, with dimensions of <100 nm [11], which are referred to as polymeric nanocomposites when made of polymer matrix and filler at the nanoscale [12]. Different NPs have been incorporated into the polymer matrix. Among these are silver (Ag) NP [13] and magnesium oxide (MgO) NP [14].

The effects of size, shape, and volume fraction of adding NPs to nanocomposite polymers on the crystallization were examined in molecular dynamics simulations. It was observed that regardless of the shape, decreasing the size of particles at the same volume fraction results in decreased final crystallinity. On the other hand, using the same particle size increases the volume fraction, resulting in a decrease in the crystal growth rate and final crystallinity. The shape of NPs has shown to have an impact on the growth rate and crystallinity, for example, using cubic particles causes a higher growth rate and crystallinity compared to spherical particles, solely due to flat surfaces of cubic NPs [15]. This is of paramount importance as it has a huge impact on chemical and mechanical properties such as stiffness and tensile strength.

Controlling the NPs’ dispersion in the polymer matrix is the most significant challenge in nanocomposite production due to the high probability of aggregation of the nanoparticles [16]. Additionally, the uniform dispersion (homogeneity) of inorganic NPs in the polymer matrix is of paramount importance when preparing polymer/inorganic nanocomposites that require enhanced physical properties, including tensile strength and impact strength [17]. Moreover, it has been reported that the more uniform NPs dispersion in the polymer causes the stronger interaction between the NPs and polymer matrix by using the particle-polymer bonding agent. For example, the surface functionalization of NPs results in the more improved mechanical properties of tensile modulus, tensile strength, and compressive modulus [18].

Several studies were conducted in which NPs were mixed with PMMA without examining the dispersion and agglomeration of NPs [11,19,20]. On the other hand, some studies have applied techniques for mixing NPs with PMMA. For example, NPs have been mixed with the PMMA powder by non-ethanol-assisted [21] or ethanol-assisted [22] as a solvent. However, none of these studies investigated the dispersion and homogeneity of NPs mixed with PMMA. Therefore, this study aimed to examine the dispersion and homogeneity of NPs mixed with PMMA by non-ethanol-assisted and non-ethanol-assisted techniques.

## 2. Materials and Methods

### 2.1. Materials

Clear orthocryl (cold cure acrylic) PMMA (powder: polymethylmethacrylate, REF 160-300-00 and liquid: methylmethacrylate, REF 161-150-00) was used in this study (DENTAURUM). In addition, Silver NP (20 nm, Spherical, 99.99%, metal basis CAS No.:7440-22-4, Hongwu International Group Ltd. Guangzhou, China), magnesium oxide NP (Mg0, 99.9%, 10–30 nm, SkySpring Nanomaterials, Inc. Houston, TX, USA), and absolute ethanol (CAS-No: 64-17-5, Darmstadt, Germany) were used.

### 2.2. Methods

#### 2.2.1. Study Groups

##### NPs (MgO and Ag) Groups

**Figure 1:**
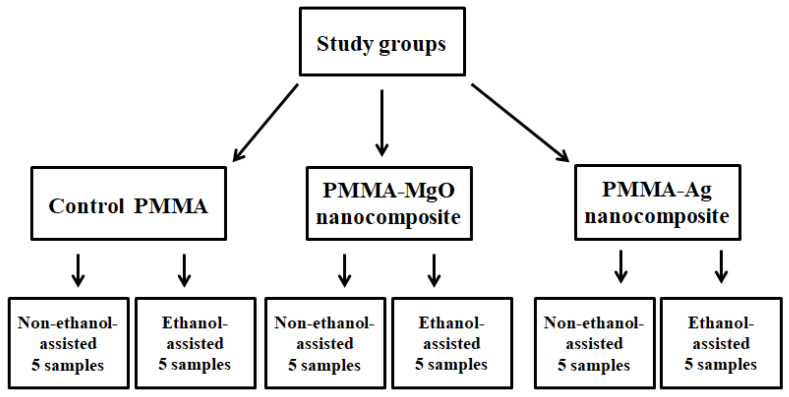
Study design and groups.

Ag and MgO NPs were mixed with PMMA powder either in non-ethanol-assisted or ethanol-assisted techniques. In general, 0.736 gm of the NPs were mixed with 10 gm PMMA powder (*w*/*w*) in order to obtain 5% PMMA-MgO and PMMA-Ag nanocomposite (Figure 1).

A. Non-ethanol-assisted mixing of NPs with PMMA powder (without ethanol)

Using sensitive balance, 0.736 gm of each of the NPs was dissolved in 10 mL absolute ethanol and then sonicated for 5 min in order to deagglomerate the NPs aggregation that might happen during transport and storage [23,24] by UP100H ultrasonic processor (Hielscher Ultrasound Technology), followed by the evaporation of absolute ethanol in the oven at 50 °C for 48 h. The remaining NPs were then ground with a mortar and pestle until a fine powder was obtained, and this was mixed with PMMA powder and stirred for 30 min at 400 rpm to obtain homogeneity [21].

B. Ethanol-assisted mixing of NP with PMMA powder (with ethanol)

As mentioned above, the sonicated NPs were dissolved in absolute ethanol and added to the PMMA powder, resulting in the formation of a viscous solution. This was followed by magnetic stirring at 400 rpm for 2 h at 50 °C until the ethanol had evaporated, and the mixed powder was then ground with a mortar and pestle. Afterwards, to ensure the evaporation of the solvent, the powder was placed in the oven at 50 °C for 4 h [22].

##### Control Group

Orthocryl (cold cure acrylic) PMMA powder was used as control after mixing in both non-ethanol-assisted and ethanol-assisted techniques with absolute ethanol, which was followed by magnetic stirring at 400 rpm for 2 h at 50 °C until the ethanol evaporated.

#### 2.2.2. Discs Preparation

Five disc samples (10 mm in diameter, 2 mm thick) for each study group were prepared using a stainless steel mold sandwiched between two glass pads [20]. PMMA powder and monomer (2.5:1 ratio) were mixed according to manufacturer’s instruction. The discs were subsequently placed in a polyclave (1 l Bu¨ chiglasuster polyclave reactor) containing water and kept under pressure of 2.2 bar at 50 °C for 25 min [25]. Furthermore, for the purpose of the Stereo microscope test, five 0.2 mm discs were prepared for each study group.

### 2.3. Dispersion and Homogeneity Tests

#### 2.3.1. X-ray Diffraction (XRD)

An X-ray diffraction machine was used to test the coating of NPs on the surface of PMMA particles and to determine the crystallite size of PMMA-NP. The required quantities of PMMA-NP nanocomposite powder for each study group (control, Ag, and MgO groups) were placed in 10 mL test tubes and desiccated in the oven at 50 °C, and then the samples were examined by XRD machine.

XRD measurements were carried out by using a PAN analytical X’ Pert PRO (Cu Kα = 1.5406 Å). The scanning rate was 1°/min in the 2θ range from 5° to 80°.

#### 2.3.2. Energy-Dispersive X-ray Spectroscopy (EDX)

EDX was used to scan PMMA-NP nanocomposite powder to identify the elemental composition of NPs over the surface of PMMA particles. In addition, it was used to detect the homogeneity and agglomeration of NP over PMMA particles.

#### 2.3.3. Scanning Electron Microscope (SEM)

To assess the dispersion and homogeneity, PMMA-NP nanocomposite powder and discs of each study group were tested using a scanning electron microscope (SEM; Quanta 450).

#### 2.3.4. Stereo Microscope

Discs with 0.2 mm thickness were evaluated for each study group under stereo microscope at 4× magnification power to assess dispersion of NPs through PMMA matrix.

## 3. Results

### 3.1. X-ray Diffraction Test

**Figure 2:**
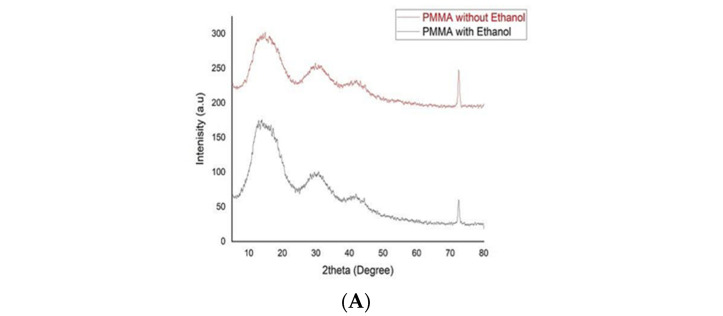

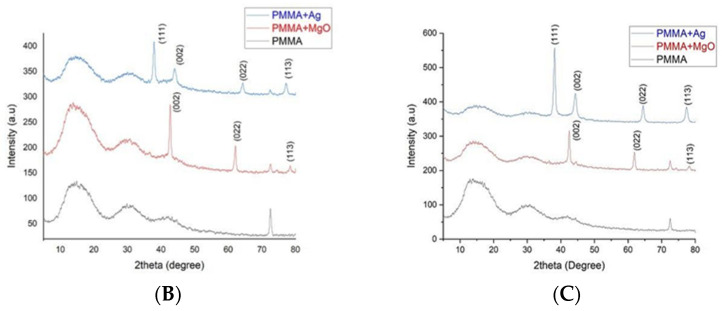
XRD test for study groups: (**A**) control group; (**B**) non-ethanol-assisted mixing; and (**C**) ethanol-assisted mixing.

The results of the XRD test for each study group are shown in Figure 1. It is apparent that there is no difference in the results between the two control groups (Figure 2A). For the MgO and Ag groups, the coating of Ag and MgO NPs over the PMMA particles is apparent with both methods (non-ethanol-assisted or ethanol-assisted mixing) (Figure 2B,C). The peaks presented in Figure 2B,C correspond to Ag NP (blue) and MgO (red), according to inorganic crystal structure database (ICSD) card No. 04-0783 and 89-7746, respectively.

By studying the full width at half maximum (FWHM) value of the XRD pattern, the crystallite size of NPs in PMMA-NP nanocomposite powder was calculated by using the following Debye–Scherrer equation:

(1) $$D=\frac{0.95\lambda }{\beta_{D}cos\theta }$$

where *D* is the average crystallite size (diameter), λ is wave length of the incident X-ray (0.154 nm), and θ is the Bragg’s angle and $\beta_{D}$ is full width at half maximum (FWHM). From the above equation, the average crystallite size was determined (Table 1). It is clear that for ethanol-assisted mixing, the average crystallite size was smaller than for non-ethanol-assisted mixing and resulted in good dispersion of NPs over the surface of PMMA particles.

### 3.2. Energy-Dispersive X-ray Spectroscopy (EDX)

**Figure 3:**
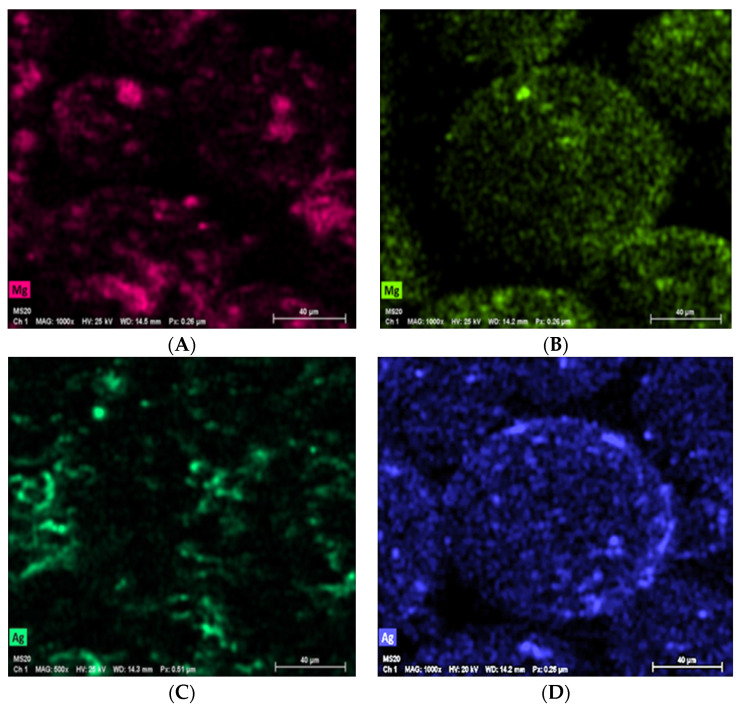
EDX photos of PMMA-MgO nanocomposite ((**A**) non-ethanol-assisted and (**B**) ethanol-assisted) and PMMA-Ag nanocomposite powder ((**C**) non-ethanol-assisted and (**D**) ethanol-assisted).

EDX spectrum photos illustrated that PMMA-MgO and PMMA-Ag nanocomposite powders mixed in ethanol-assisted method have better attachment and good dispersion over the surface of PMMA particles, with less NPs agglomeration compared with the non-ethanol-assisted mixing method, i.e., in the ethanol-assisted method, NPs followed the shape of PMMA particles, and the color shade of NPs was evenly dispersed without the dark- and light-colored areas on PMMA particles as present in non-ethanol-assisted method, which indicates the state of NPs agglomeration (Figure 3).

### 3.3. Scanning Electron Microscope (SEM)

**Figure 4:**
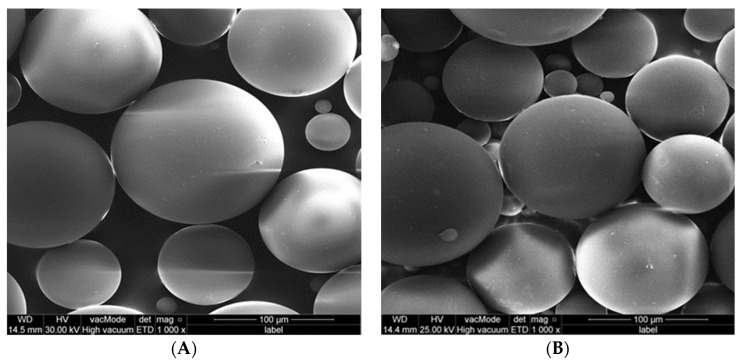
SEM for powder of control group, (**A**) PMMA powder alone; (**B**) PMMA powder with ethanol.

**Figure 5:**
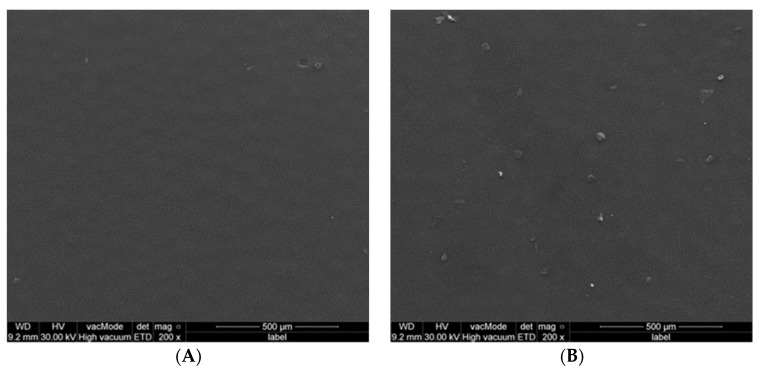
SEM for disc of control group, (**A**) PMMA alone; (**B**) PMMA with ethanol.

SEM analysis revealed that the average particle size of the PMMA powder was approximately 50 µm, within a range of 10 µm to 100 µm. Adding ethanol resulted in no difference in control group (Figure 4). Additionally, no differences were detected in the surface morphology of PMMA disc samples (PMMA alone and with ethanol) (Figure 5).

**Figure 6:**
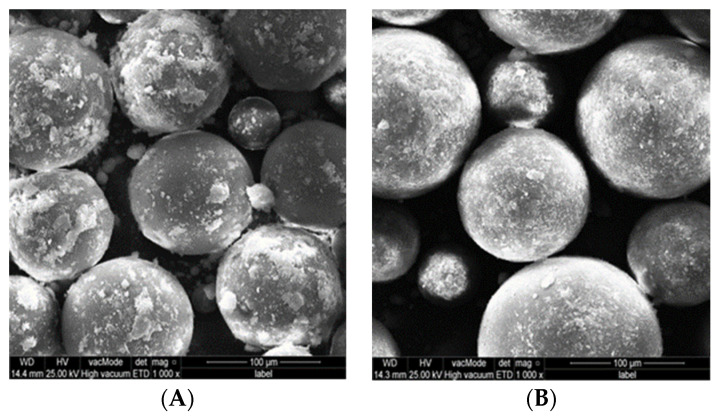

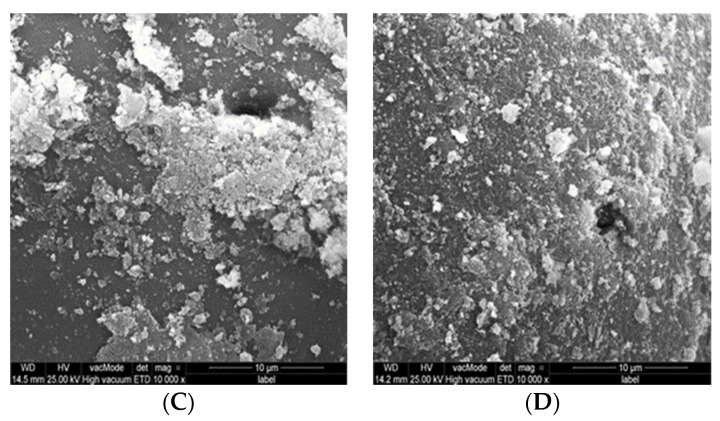
SEM photos of PMMA-MgO nanocomposite powder after non- ethanol-assisted (**A**,**C**) and ethanol-assisted (**B**,**D**) mixing.

**Figure 7:**
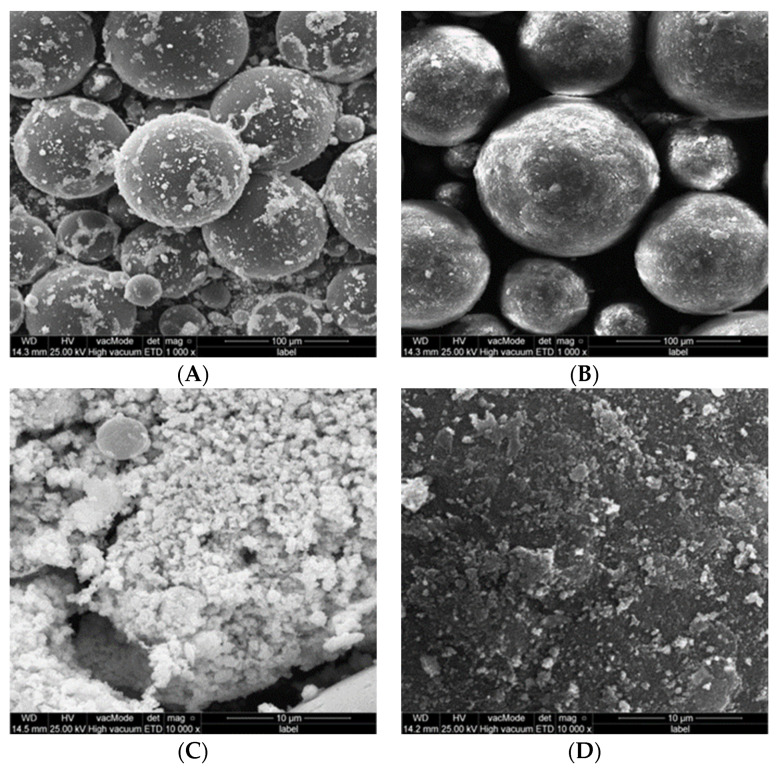
SEM photos of PMM-Ag powder after non-ethanol-assisted (**A**,**C**) and ethanol-assisted (**B**,**D**) mixing.

SEM of 5% PMMA-MgO nanocomposite (Figure 6) and 5% PMMA-Ag nanocomposite (Figure 7) powder showed that non-ethanol-assisted mixing resulted in no attachment of NPs to the surface of PMMA particles and a lot of NPs agglomeration; this is easily noticed by observing NPs agglomeration between PMMA particles, whereas in ethanol-assisted mixing, NPs with PMMA showed better attachment of NPs to the surface of PMMA powder particles and good dispersion of NPs over the surface of PMMA particles, based on an absence of freely flowing NPs aggregation between PMMA particles in comparison to the non- ethanol-assisted mixing.

**Figure 8:**
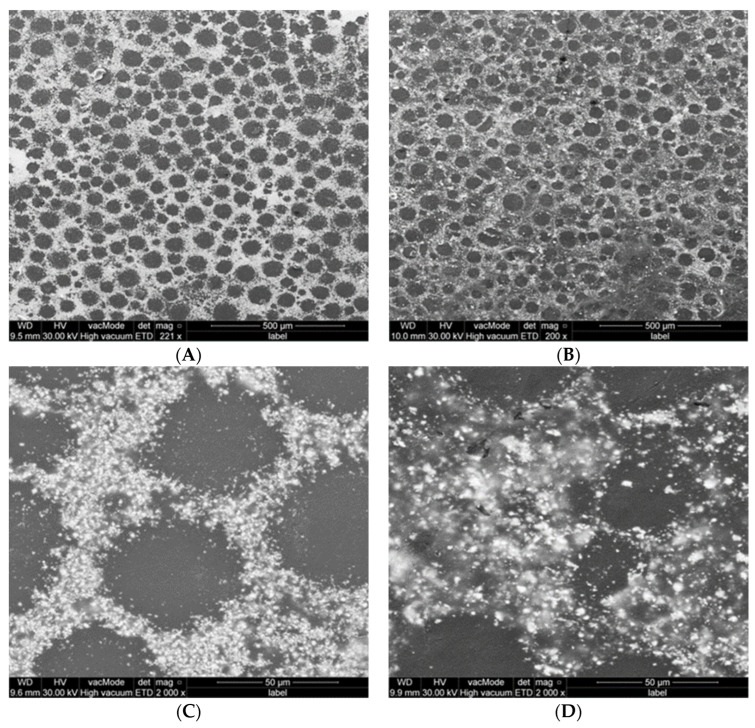
SEM photos of PMMA-MgO nanocomposite discs prepared by non-ethanol-assisted (**A**,**C**) and ethanol-assisted (**B**,**D**) mixing.

**Figure 9:**
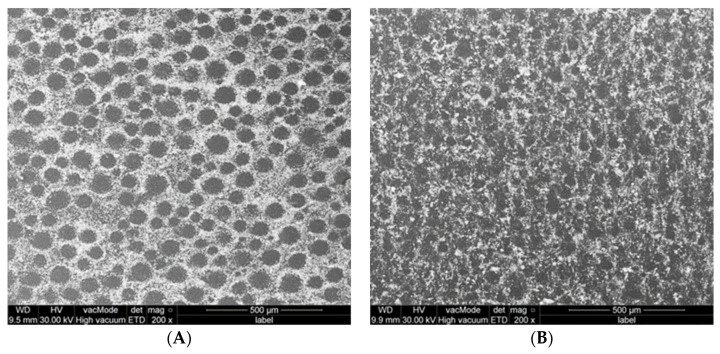

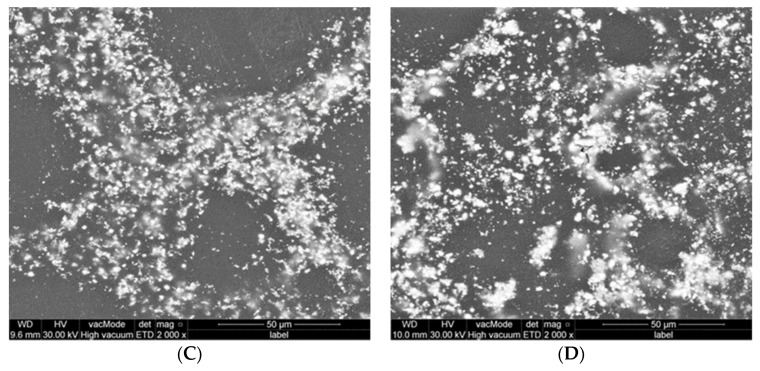
SEM photos of PMMA-Ag nanocomposite discs (**A**,**C**) prepared by non- ethanol-assisted mixing and (**B**,**D**) by ethanol-assisted mixing.

Regarding the discs of PMMA-MgO and PMMA-Ag nanocomposite, the good dispersion and homogeneity of NPs in the matrix of PMMA was again noticed, with the least NPs agglomeration from ethanol-assisted mixing compared to the non-ethanol-assisted mixing (Figure 8 and Figure 9); this can be observed through clear outlined PMMA particles without the absorption of NPs as in A and C in Figure 8 and Figure 9.

### 3.4. Stereo Microscope Analysis

**Figure 10:**
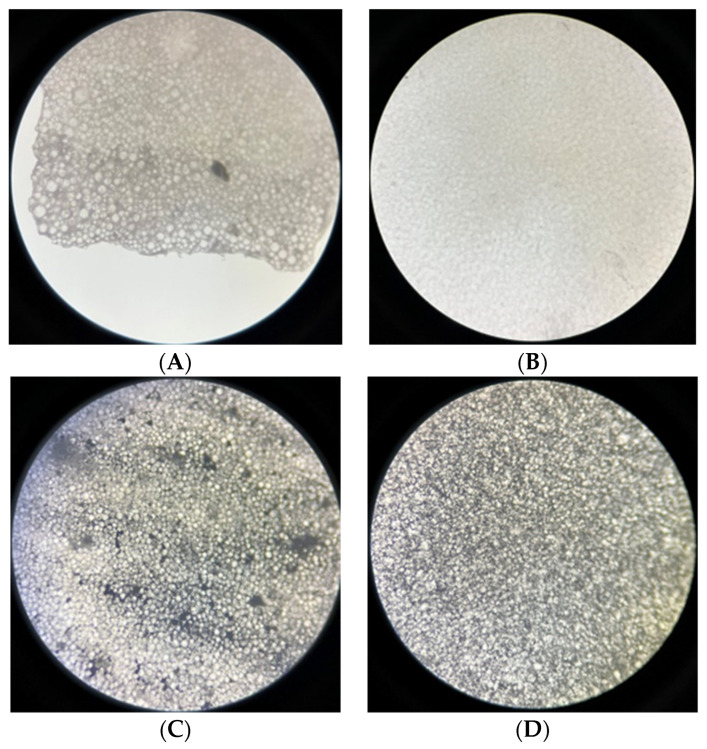
Stereo microscope photos of PMMA-MgO nanocomposite disc ((**A**) non-ethanol-assisted and (**B**) ethanol-assisted) and PMMA-Ag nanocomposite disc ((**C**) non-ethanol-assisted and (**D**) ethanol-assisted).

Again, the stereo microscope analysis of MgO-PMMA and Ag-PMMA nanocomposite discs (0.2 mm thickness) showed that the ethanol-assisted mixing of NPs with PMMA resulted in better dispersion and no agglomeration of NPs within the PMMA matrix when compared to non-ethanol-assisted mixing (Figure 10).

## 4. Discussion

Many types of polymers are reinforced with NPs [26] to enhance the physical properties of PMMA. In recent years, metal oxide NPs have been largely investigated for their activity as antimicrobial additives [19].

PMMA is among the most commonly used dental materials. It is mainly used for the fabrication of acrylic denture bases including removable orthodontic appliances, with successful results attributed to its optimal esthetics and biocompatibility. However, its poor mechanical properties, particularly its low tensile and impact strength, are among its major drawbacks, potentially leading to easy fracture or cracking [27]. This is especially the case with cold-cure acrylic resins that have a higher porosity than heat-cure acrylic resins [28]. The incorporation of NPs into acrylic resin powder was recently suggested to improve its mechanical properties [29] as well as its antimicrobial properties [30].

Ethanol has been commonly used as stabilizer because it is a polar solvent with the OH group that reacts with ions; thus, dispersion in ethanol lasts longer. Furthermore, dispersing PMMA in ethanol provides more surface area for NPs to be absorbed by the PMMA particles. It is worth mentioning that in this technique, the dispersion of inorganic NPs on PMMA is based on the electrostatic interactions that occur between the negatively charged PMMA particles and the positively charged NPs (MgO and Ag). This technique, called noncovalent attachment, can be carried out via the dispersion of NPs and the polymer in a media (ethanol in the current study) [31,32]. However, other techniques such as adding surfactants or coupling agents in NPs and PMMA mixing provide the addition of functional groups to the surface of NPs, allowing conventional chemical reactions with the PMMA polymer, which leads to better attachment of NPs over the surface of PMMA particles [33].

In the present research, the two common methods of mixing of NPs with PMMA powder were used: non-ethanol-assisted mixing and stirring [34] and ethanol-assisted mixing with aid of absolute ethanol solvent [22]. Although several studies have investigated the impact of adding NPs to PMMA on the mechanical properties of the PMMA-NP nanocomposite, no studies have tested the dispersion and homogeneity of NPs within PMMA. It is apparent that the agglomeration of NPs within the PMMA matrix unfavorably affects the reaction of monomers, leading to increased levels of unreacted monomer, which acts as a plasticizer [35,36]. The increase in NPs content causes these particles to agglomerate, and the agglomerated NPs can act as stress-concentrating centers in the matrix and adversely affect the mechanical properties of the polymerized material [37]. It is important to acknowledge that as the study examined the NPs, it would have been better to have tested on a nanoscale. However, in the present study the dispersion of NPs on PMMA particles was considered to be assessed on a microscale because the average PMMA particles was 50 microns.

Shahabi et al. evaluated the effects of the non-ethanol-assisted mixing of chitosan NPs with PMMA on mechanical properties, and it was found that the flexural strength, compressive strength, microhardness, and impact strength of the PMMA–chitosan nanocomposite decreased in comparison to the control group [38]. This could be associated with the dispersion and agglomeration of NPs on the PMMA surface. The current study used four tests to determine the impact of two commonly used mixing methods of NPs with PMMA on dispersion and homogeneity. To the best of our knowledge, this is the first study to test the dispersion and homogeneity of mixing NPs with PMMA. Having well dispersed and homogenic mixing of any mixing particles is of paramount importance for enhancing their mechanical properties.

The introduction of nanoparticles in ethanol at low temperatures in order to obtain a nanocomposite plays an important role in deagglomeration and dispersion of the NPs [39]. The better dispersion and homogeneity achieved by the ethanol-assisted method of mixing might be related to the crystallite size of the NPs in the PMMA-NP nanocomposite. It is obvious that smaller crystallite size leads to less agglomeration and better dispersion [40]. This is in accordance with the finding by the current study in XRD analysis that regarding the PMMA-MgO nanocomposite and PMMA-Ag nanocomposite, the crystallite size of NPs was smaller after mixing in ethanol-assisted technique, and consequently better dispersion and homogeneity were then obtained when compared to the non-ethanol-assisted mixing of NPs with PMMA powder.

The examination of other NPs, such as calcium β-pyrophosphate and zinc oxide, by non-ethanol-assisted mixing with PMMA and SEM revealed that the non-ethanol-assisted mixing of NPs with PMMA is associated with non-homogeneity and poorly dispersed NPs over PMMA [41,42]. These results are commensurate with the result of this study and highlight the importance of using ethanol as a solvent for mixing NPs with PMMA. On the other hand, another study found that mixing metal sulfide NPs with PMMA by toluene solvent obtained good dispersion of the metal sulfide nanoparticles into PMMA matrices [43]. This is in line with the result of the current study, thus confirming the importance of solvent solution for the mixing of NPs with PMMA.

This study only examined the effect of ethanol on the mixing of MgO and Ag with PMMA, and further studies are required to check the role of ethanol on mixing other NPs with PMMA and other polymers. Further analysis such as transmission electron microscopy is recommended to compare the size and morphological changes of NPs before and after mixing with PMMA. This study has the limitations that only two NPs were examined and that the mechanical properties have not yet been examined in relation to each corresponding technique. Nonetheless, the results of this study provide valuable pointers for consideration in the future.

## 5. Conclusions

The results of this study suggest that using ethanol as a solvent for mixing MgO and Ag NPs with PMMA microparticles obtains better dispersion and homogeneity, which ultimately might result in better mechanical properties of modified acrylic resin (PMMA-NP nanocomposite). Further studies are recommended to test the mixing efficacy of these NPs with PMMA in a melting state and nanoscale.

## Figures and Tables

**Table 1:** Crystallite size of PMMA nanocomposite powders of Ag and MgO after non-ethanol-assisted mixing and ethanol-assisted mixing (sample number = 5 per each group).

Sample	Position (2theta)	Planes	Non-Ethanol-Assisted		Ethanol-Assisted	
			FWHM Left [°2Th]	Size (nm)	FWHM Left [°2Th]	Size (nm)
PMMA	72.49		0.288	34.2	0.288	34.2
PMMA-MgO group	42.765	(002)	0.315	27.1	0.315	27.1
	62.135	(022)	0.236	39.3	0.236	39.3
	72.59	(113)	0.394	25.0	0.709	14.5
Average			0.315	30.46	0.420	26.96
PMMA- Ag group	38.01	(111)	0.315	26.7	0.315	26.7
	44.139	(002)	0.551	15.6	0.63	13.6
	64.355	(022)	0.315	29.8	0.472	19.9
	77.26	(113)	0.394	25.8	0.63	16.1
Average			0.3937	24.47	0.5117	19.07

## Data Availability

The data that support the findings of this study are available from the corresponding author upon reasonable request.
